# Identification of Signatures of Selection by Whole-Genome Resequencing of a Chinese Native Pig

**DOI:** 10.3389/fgene.2020.566255

**Published:** 2020-09-17

**Authors:** Wei Zhang, Min Yang, Mei Zhou, Yuanlang Wang, Xudong Wu, Xiaodong Zhang, Yueyun Ding, Guiying Zhao, Zongjun Yin, Chonglong Wang

**Affiliations:** ^1^Key Laboratory of Pig Molecular Quantitative Genetics of Anhui Academy of Agricultural Sciences, Anhui Provincial Key Laboratory of Livestock and Poultry Product Safety Engineering, Institute of Animal Husbandry and Veterinary Medicine, Anhui Academy of Agricultural Sciences, Hefei, China; ^2^College of Animal Science and Technology, Anhui Agricultural University, Hefei, China; ^3^Faculty of Animal Science and Technology, Yunnan Agricultural University, Kunming, China

**Keywords:** Anqing six-end-white pig, SNP, signatures of selection, *F*_*ST*_, θπ

## Abstract

Identification of genomic signatures of selection that help reveal genetic mechanisms underlying traits in domesticated pigs is of importance. Anqing six-end-white pig (ASP), a representative of the native breeds in China, has many distinguishing phenotypic characteristics. To identify the genomic signatures of selection of the ASP, whole-genome sequencing of 20 ASPs produced 469.01 Gb of sequence data and more than 26 million single-nucleotide polymorphisms. Combining these data with the available whole genomes of 13 Chinese wild boars, 157 selected regions harboring 48 protein-coding genes were identified by applying the polymorphism levels (θπ) and genetic differentiation (*F*_*ST*_) based cross approaches. The genes found to be positively selected in ASP are involved in crucial biological processes such as coat color (**MC1R**), salivary secretion (**STATH**), reproduction (**SPIRE2**, **OSBP2**, **LIMK2**, **FANCA**, and **CABS1**), olfactory transduction (**OR5K4**), and growth (**NPY1R**, **NPY5R**, and **SELENOM**). Our research increased the knowledge of ASP phenotype-related genes and help to improve our understanding of the underlying biological mechanisms and provide valuable genetic resources that enable effective use of pigs in agricultural production.

## Introduction

Until the Neolithic Age about 10,000 years ago, humans have changed from nomadic to settled, which make captive breeding possible, and the wild boars were successfully domesticated by human at multiple locations around the world (Giuffra et al., 2000; Kijas and Andersson, 2001; Larson, 2005). Selective breeding has generated approximately 300 pig breeds that are adapted to various environmental conditions and production system (Veirano Fréchou, 2007). Natural and artificial selection played a key role in shaping the fitness of domestic pig to those environments and demands, with the underlying mechanisms being of great interest in evolutionary biology, including the relationship between molecular and phenotypic changes and how the involvement of natural and artificial processes in the evolutionary process have shaped the modern animal genomes.

According to the theory of population genetics, the functional genes subject to selection would reveal characteristic patterns due to selection preference, and these patterns are known as “signatures of selection” (Fan et al., 2014). The selected region is often a chromosomal region with low genetic diversity within the group and high genetic differentiation rate between groups. A series of statistical approaches, based on the genetic diversity and genetic differentiation, have been proposed for the detection of selection signatures, such as genetic differentiation (*F*_*ST*_) and polymorphism levels (θπ, pairwise nucleotide variation as a measure of variability). The fixation index (*F*_*ST*_) statistic, which is based on population differentiation, was first defined by Lewontin and Krakauer (1973) based on coefficient *F* (Wright, 1949) and developed by Weir and Cockerham (1984), Akey et al. (2002), and Gianola et al. (2010). The polymorphism levels (θπ) statistic, which is based on genetic diversity, was measured for each individual by nucleotide diversity π (Nei and Li, 1979) and Watterson’s estimator θ (Watterson, 1975) and usually used to identify regions of selection between domesticated and wild species. Surveying the genomic regions with θπ and F_*ST*_ based cross approaches is useful for the detection of selection signatures (Li et al., 2013).

Recent whole genome-wide scans in diverse pig breeds aimed to uncover the underlying mechanism for complex phenotype–genotype association showed that detecting comprehensive signatures of intense artificial selection could acknowledge the characteristics of well-defined breeds (Groenen et al., 2012; Rubin et al., 2012; Li et al., 2013; Frantz et al., 2015). For example, the selected genes *NR6A1*, *PLAG1*, and *LOCRL* have played a key role on elongation of body length in European domestic pigs. Fang et al. (2009) elucidated that coat color variation is the result of intentional selection at the porcine melanocortin receptor 1 (*MC1R*) locus (Fang et al., 2009). A study identified genomic selection regions at *HIFA*, a master regulator of oxygen homeostasis, in Tibetan wild boar (Li et al., 2013). Ai et al. (2015) and Chen et al. (2018) identified that copy number variation in the *MSRB3* could regulate the size of porcine ear and identified that the *TGFB3* and *DAB2IP* played an important role in regulating the number of ribs (Zhu et al., 2015; Chen et al., 2018). Although many studies have been done in various pig breeds, in view of the diverse phenotypes among the 300 pig breeds, efforts still need to be done in elucidating the phenotype differences resulting from different environments and artificial selection.

Anqing six-end-white pig (ASP) is a representative Chinese indigenous, disease-resistant breed with high fertility, high fat content, excellent meat quality, good maternal stability, and a crude-feed tolerance that has been bred with artificial selection for a long time. In our previous studies, the average backfat thickness and the intramuscular fat content in the longissimus dorsi muscle of ASP at about 100 kg were measured, and the value were 46.01 ± 2.55, 6.54 ± 0.81 (mean ± SD, *n* = 6) separately (Wang et al., 2020). Based on molecular and multiomics level, we have identified some genes, microRNAs (miRNAs) and long non-coding RNAs (lncRNAs) played an important role in meat quality, lipid metabolism, and fat deposition (Zhang et al., 2015; Hu et al., 2019; Ding et al., 2020; Wang et al., 2020). However, the genetics basis of the characteristics in ASP, particularly at the genomic level, remains largely unknown.

To access a comprehensive analysis of genetic variations underlying domestication traits in ASP breed, we used whole-genome resequencing data of 20 unrelated ASP, together with 13 publicly available Asian wild boar genomes, using the *F*_*ST*_ and θπ based cross approaches to explore the signatures of selection in ASP. In this study, we identified a suite of genes having undergone positive selection that may contribute to domestication phenotypes, including disease resistance, reproduction, digestive system, and lipid metabolism. The findings herein will provide insights to increase understanding of the genetic basis that determines the unique traits of ASP and provide scientific foundation for its development and utilization.

## Materials and Methods

### Sample Collection, DNA Extraction, and Sequencing

**FIGURE 1:**
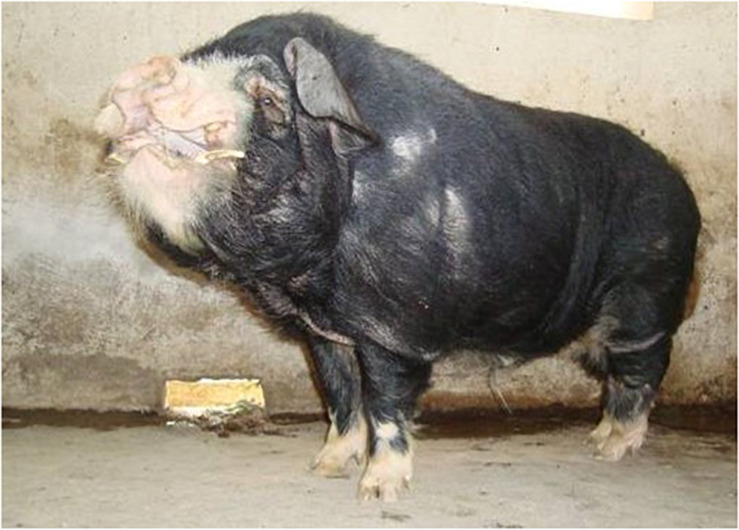
Anqing six-end-white pig used in this study.

We sampled 20 unrelated ASP (Figure 1), collecting from the ASP conservation farm (Anqing, China; longitude, 116°33′E; latitude, 30°19′N). Genomic DNA was extracted from the ear samples using a standard phenol–chloroform method (Sambrook and Russell, 2001) and was stored at 4°C to avoid freeze–thawing and tested for concentration (as ng/μl) using a Nanodrop. The DNA was fragmented and treated following the Illumina DNA sample preparation protocol, with a process of end-repaired, A-tailed, ligated to paired-end adaptors and PCR amplification with 350-bp inserts. The constructed libraries were sequenced on the Illumina HiSeq X Ten platform (Illumina, San Diego, CA) for 150-bp paired-end reads at Novogene (Beijing, China). The raw resequencing reads were filtered using NGSQCToolkit, which removed reads containing adapter or poly-N, low-quality reads with >30% base having Phred quality ≤20, the 5′ and 3′ ends 5 bp low-quality base of a read.

To comprehensively investigate the signatures of selection of ASP population during domestication and breeding, we also retrieved 13 resequencing data of Asian wild boars (∼13× coverage per individual) using as reference group (Supplementary Table S1) (Groenen et al., 2012; Li et al., 2013; Ai et al., 2015).

### Variant Calling and Annotation

The filtered resequencing reads from all genome were then mapped independently to pig reference genome using BWA-MEM version 0.7.10 (Li and Durbin, 2009) with default parameters. We also downloaded the reads file for 13 pig individuals from National Center for Biotechnology information (NCBI) under BioProject ID ERP001813 and PRJNA260763 and processed these data with the same pipeline. The variation detection followed the best practice workflow recommended by GATK. HaplotypeCaller and GenotypeGVCFs algorithms were jointly used to call variants. Intermediate genomic (gVCF) file were generated using the “-ERC GVCF” mode in “HaplotypeCaller.” Joint genotype was performed using “GenotypeGVCFs” and then subsequently merged with BCFtools (Li et al., 2009). We adopted following hard filtering criteria to retain high-quality variations with “Depth >4.0, MQ RMS mapping quality >20 and -cluster 2, -window 4” for SNPs: “QD <2.0 || FS >200.0 || ReadPosRankSum <–20.0” for insertions/deletions (InDels). After filtering, the variants were annotated with the ANNOVAR software based on gene- and region-based model (Wang et al., 2010).

### Population Genetic Structure and Linkage Disequilibrium

To investigate the genetic relationships between ASP and Asian wild pig population, we filtered all autosome single nucleotide variants (SNVs) with a minor allele frequency (MAF) <0.05 and linkage disequilibrium (*r*^2^) <0.2, site missing rate <0.05, and quality value <30 for principal component analysis (PCA), neighbor-joining phylogenetic trees, and linkage disequilibrium; and – indep-pairwise 100 10 0.01 using plink 1.9 for structure. Neighbor-joining (NJ) phylogenetic trees were built based on identical-by-state distance matrix using the PHYLIP v.3.695 package (Felsenstein, 1989). In addition, PCA analyses were performed using the GCTA software (v.1.25) (Yang et al., 2011), and the first three eigenvectors were plotted. Moreover, the population ancestry was inferred by ADMIXTURE (v1.3.0) (Holsinger and Weir, 2009) with a fast-maximum likelihood method. The optimum number of ancestral clusters K was estimated with the five-fold cross-validation procedure. The genome-wide linkage disequilibrium (LD) pattern between ASP and AWB population were assessed using the PopLDdecay software^1^ to calculate the average *r*^2^ value with the default parameters.

### Genome-Wide Selective Sweeps Detection

Before performing detecting signatures of selection, we filtered the SNVs with call rates <0.90 and MAF <0.05 and remove sites with a missing rate >20% using VCFtools. Even though our Asian wild boars may not fully reflect the genetic diversity of the true progenitor wild Asian pig population, we decided to identify some potential selective signals during pig domestication (ASP versus Asian wild boar) by surveying the genomic regions with polymorphism levels (θπ, pairwise nucleotide variation as a measure of variability) and genetic differentiation (*F*_*ST*_) based cross approaches, using a 100-kb sliding window approach with 10 kb step size to calculate *F*_*ST*_ and θπ values with PopGenome (Pfeifer et al., 2014). The overlapped windows within the top 1% of the *F*_*ST*_ and θπ ratio empirical distributions were considered as the candidate selective regions and were subsequently examined for the candidate genes (Li et al., 2013).

### Annotation of the Selected Genes and Quantitative Trait Loci Analysis

To further explore the potential biological significance of genes within these sweep regions, Gene Ontology (GO) terms and Kyoto Encyclopedia of Genes and Genomes (KEGG) Pathway Enrichment analyses were carried out through the Database for Annotation, Visualization and Integrated Discovery (DAVID, v.6.8) (Huang et al., 2009). Only terms with a *p* < 0.05 were considered to be significant. Additionally, the pig quantitative trait loci (QTL) database^2^ was used to annotate potential traits related to the potential selection regions based on the physical position of the QTLs.

### Protein–Protein Interaction Network Analysis of the Selected Genes

To investigate the interaction associations of selected genes, we applied the selected genes to the Search Tool for the Retrieval of Interacting Genes (STRING,^3^) (Szklarczyk et al., 2015), a tool to retrieve and display the genes a query gene repeatedly occurs with in clusters on the genome. The selected genes were mapped and the interactions with Default confidence cutoff of 400 was used. Afterward, a protein–protein interaction (PPI) network was constructed and visualized by Cytoscape software (version 3.4.0,^4^).

## Results

### Genomic Variant Identification in ASP Breed

To detect genome-wide variation in ASP breed, we performed whole-genome resequencing of 20 unrelated ASPs, which yield 469.01 Gb of sequence data with an average depth of 9× (Table 1). The data were uploaded to the NCBI with BioProject ID (PRJNA634804). After variants calling and subsequent stringent quality filtering, a total of ∼26 million SNVs with high quality were finally retained, of which 391,337 SNVs were newly identified (not included in the dbSNP database:^5^). These novel SNVs were expected to be present at lower frequencies or to be specific to the ASP population, accounting for their lack of previous detection. For all detected SNVs, the average transition/transversion ratio was 2.29, concordant with the previous report (Kerstens et al., 2009).

**TABLE 1:** Summary statistic of resequencing data.

Sample	Raw reads	Effective rate (%)	Raw base (G)	Clean base (G)	Coverage	Coverage at least 1 or 4 × (%)
S19	83,823,171	99.7	25.15	25.07	9.64	98.90 or 95.33
S9	86,473,158	99.85	25.94	25.90	9.96	99.02 or 94.58
S18	72,934,572	99.72	21.88	21.82	8.39	98.69 or 92.56
S1	75,402,685	99.79	22.62	22.57	8.68	99.88 or 92.05
S13	82,954,876	99.71	24.89	24.81	9.54	98.72 or 94.99
S12	79,354,645	99.57	23.81	23.70	9.12	98.71 or 93.83
S15	76,047,672	99.74	22.81	22.75	8.75	98.67 or 93.36
S14	88,777,383	99.73	26.63	26.56	10.22	98.80 or 96.13
S17	81,000,552	99.71	24.30	24.23	9.32	98.74 or 94.64
S16	82,788,651	99.73	24.84	24.77	9.53	98.76 or 95.00
S10	77,587,033	99.75	23.28	23.22	8.93	99.01 or 93.02
S20	83,019,325	99.71	24.91	24.83	9.55	98.68 or 94.91
S7	71,153,986	99.84	21.35	21.31	8.20	98.83 or 90.59
S5	71,392,366	99.83	21.42	21.38	8.22	98.78 or 90.32
S11	75,019,716	99.72	22.50	22.43	8.63	98.62 or 92.79
S8	74,686,600	99.84	22.40	22.37	8.60	98.85 or 91.78
S6	75,178,013	99.81	22.55	22.51	8.66	98.95 or 92.17
S4	75,380,120	99.83	22.61	22.57	8.68	98.94 or 92.05
S2	75,927,310	99.82	22.78	22.73	8.74	98.90 or 92.16
S3	74,458,919	99.83	22.34	22.30	8.58	98.91 or 91.96

Further annotation of these SNVs in the ASP population revealed that they were most abundant in intergenic regions (59.89%) and intronic regions (37.02%), followed by downstream (0.58%), upstream (0.57%), untranslated regions (1.14%), and splicing sites; only 0.81% were located in coding sequences. Of the SNPs present in coding regions, 140,436 were synonymous and 72,779 were non-synonymous (Table 2).

**TABLE 2:** Annotation of the single nucleotide variants (SNVs).

Variant type	No. of variants
SNV	26,401,669
Intergenic	15,811,056
downstream	153,338
upstream	149,936
5′UTR	55,005
3′UTR	244,339
Splicing site	1,002
Intron	9,772,851
Coding domain	214,142
Synonymous	140,436
Non-synonymous	72,779

### Population Genetic Structure and Linkage Disequilibrium

**FIGURE 2:**
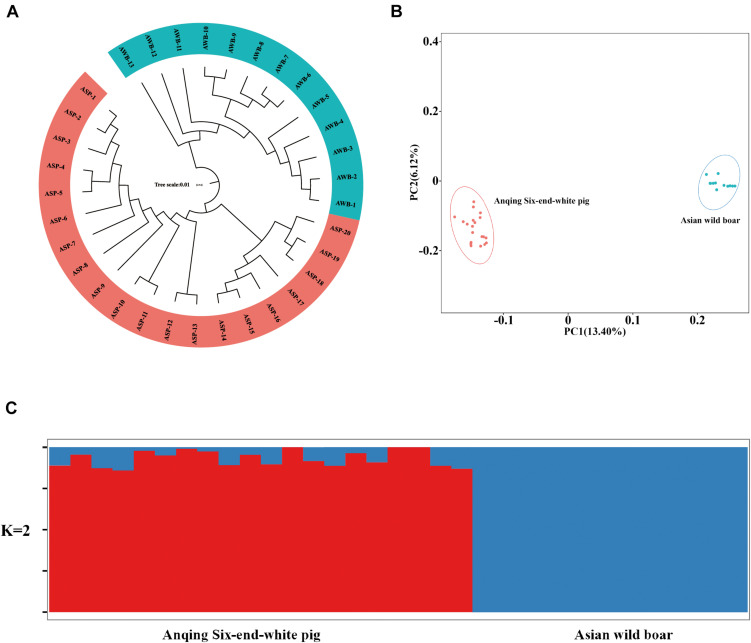
Population structure and principle component analysis. **(A)** Neighbor-joining tree constructed from single nucleotide variant (SNV) data among study population. **(B)** Principle component plots for the first two PCs for all 33 individuals. **(C)** Structure analysis on all the Asian wild boars and Anqing six-end-white pigs with *K* = 2.

**FIGURE 3:**
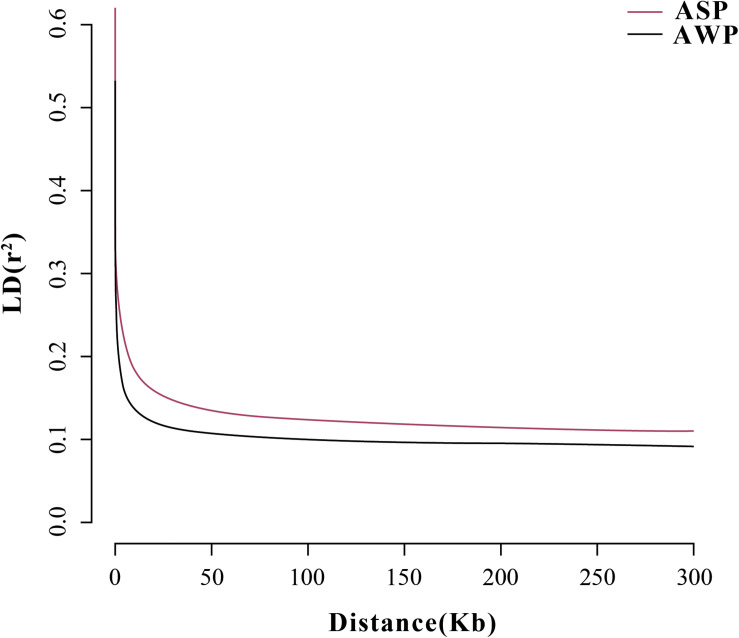
Correlation coefficients (*r*^2^) were calculated for the Asian wild boar and Anqing six-end-white pig over 50-kb windows.

After filtering, there are 201,945 SNVs for structure analyses and 18,859,059 SNVs for PCA, LD, and phylogenetic trees analyses. To assess the phylogenetic relationship among the pig breeds in this study, unrooted phylogenetic tree analyses revealed genetically distinct cluster according to their type (Figure 2A). The branches of the phylogenetic tree were grouped as expected and were consistent with the results of PCA (Figure 2B), thus revealing clustering into two distinct genetic groups. We performed PCA using GCTA, the same type cluster together. The first two PCs explain 13.4 and 6.12% of the total variation, respectively. To further understand the degree of admixture in the population, *K* = 2 was used. As shown in Figure 2C, it can separate all of the ASPs from Asian wild boars. Using the phased genotypes, linkage disequilibrium, in terms of the correlation coefficient (*r*^2^), was calculated for Anqing six-end-white and the Asian wild pig populations. As shown in Figure 3, the LD decay rates were similar between ASP and AWB populations. The faster LD decay was observed in the AWB population, which indicates that artificial selection can facilitate the increase in LD within a population.

### Identification of Selective Loci

**FIGURE 4:**
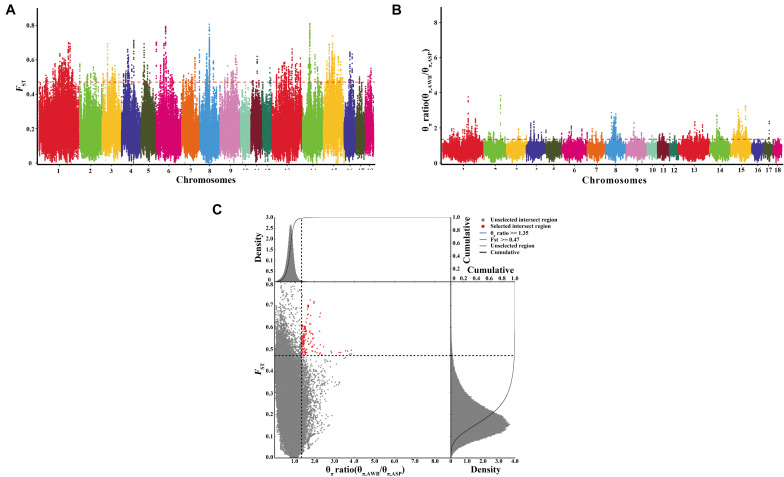
Identification of genomic regions with strong selective sweep signals in Anqing six-end-white pig population. Distribution of θπ ratio (θ_π, AWB_/θ_π, ASP_) and *F*_*ST*_, which are calculated in 100-kb windows sliding in 10-kb steps. **(A)** Distribution of *F*_*ST*_ values among autosome chromosomes. The red line represents the 0.01 level. **(B)** Distribution of θπ ratio among autosomal chromosomes. The red line represents the 0.01 level. **(C)** The final selection regions based on two statistics. Points located to the right of the vertical dashed lines (corresponding to 1% right tails of the empirical θπ ratio distribution, where θπ ratio is 1.352358) and above the horizontal dashed line (1% above tail of the empirical *F*_*ST*_ distribution, where *F*_*ST*_ is 0.470988) were identified as selected regions for Anqing **s**ix-end-white pig (ASP) (red points).

After filtering, there are 21,637,726 SNVs used for signatures of selection detection. We used both the polymorphism levels (θπ, pairwise nucleotide variation as a measure of variability) and genetic differentiation (*F*_*ST*_) based cross approaches to investigate the selection signals across the whole genome. In this study, we selected regions both meeting the top 1% threshold as the selected regions. There are 2,264 selective regions for each approach, which covered 10% of the genome. The genome distribution of the two statistics are shown in Figures 4A,B. There are 157 selected regions (15.7 Mb of the genome, Figure 4C and Supplementary Table S2) with extremely high *F*_*ST*_ values and significantly high θπ ratios, which meet both top 1% of two values (threshold, 1%; *F*_*ST*_, 0.470988; θπ ratio, 1.352358). On average, there are 70 SNVs in each window. A total of 48 genes harbored in these regions (Supplementary Table S3). Further, the SNPs in the selected genes were extracted. There is a total of 25,077 SNVs in the 48 genes, and they were most abundant in intronic regions (94.7%), followed by untranslated regions (2.93%), and only 2.32% were located in coding sequences. Of the SNPs present in coding regions, 338 were synonymous and 245 were non-synonymous (Supplementary Table S4). Thirty synonymous were randomly selected and validated by Sanger sequencing. The synonymous and primer information were shown in Supplementary Tables S5, S6 separately. The results of Sanger sequencing were consistent with the whole-genome resequencing.

### Annotation of the Selected Genes and Quantitative Trait Loci Analysis

**FIGURE 5:**
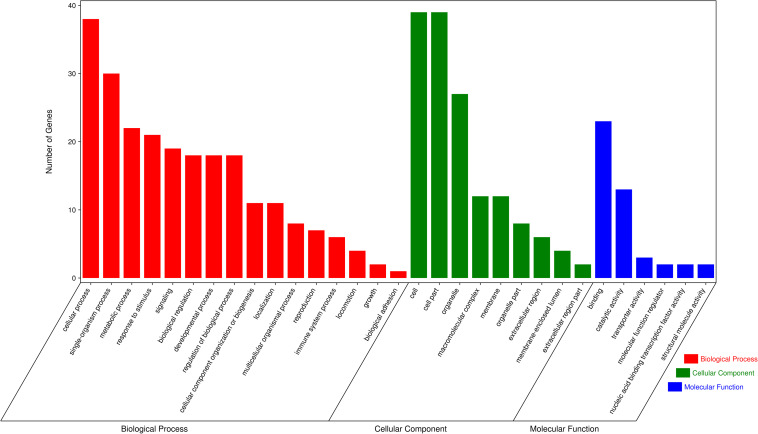
Gene Ontology (GO) terms of the selected genes, red referring to biological process, green referring to cellular component and blue referring to molecular function.

**FIGURE 6:**
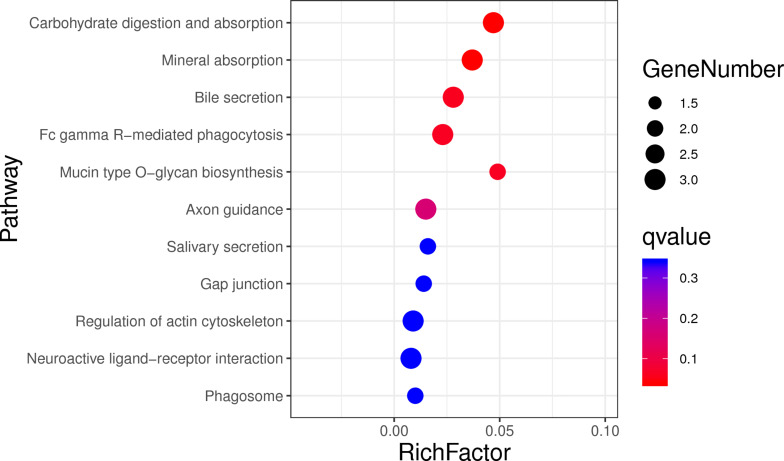
Top 20 enrichment pathways, the size of dot refers to the number genes related to pathway, and the red to blue indicate the significant *q*-value change.

In order to assess the function of these genes, GO and KEGG analyses were conducted. After enrichment analysis of the selected genes, there are 56 cellular component term, 44 molecular function terms, and 107 biological process terms. In the GO term level 2, 31 GO terms were enriched (Supplementary Table S7). Most of these genes were related to reproduction (7 genes), immune system process (6 genes), growth (3 genes), and response to stimulus (18 genes) (Figure 5). In the KEGG analysis, a total of 14 pathways were enriched, and some important pathways were also found, although there is only one gene in the pathway (Supplementary Table S8). Most of pathways were related to carbohydrate digestion and absorption (three genes), salivary secretion (two genes), olfactory transduction (one gene), peroxisome proliferator-activated receptor (PPAR) signaling pathway (one gene), and MAPK signaling pathway (one gene) (Figure 6). We also found a selected gene (MC1R), which could explain the coat color phenotype of ASP. QTL overlapping with the potential selection regions detected by two methods was associated with meat and carcass (45.97%), reproduction association (11.09%), production (10.42%), and so on, as shown in Supplementary Table S9.

### PPI Network Construction

**FIGURE 7:**
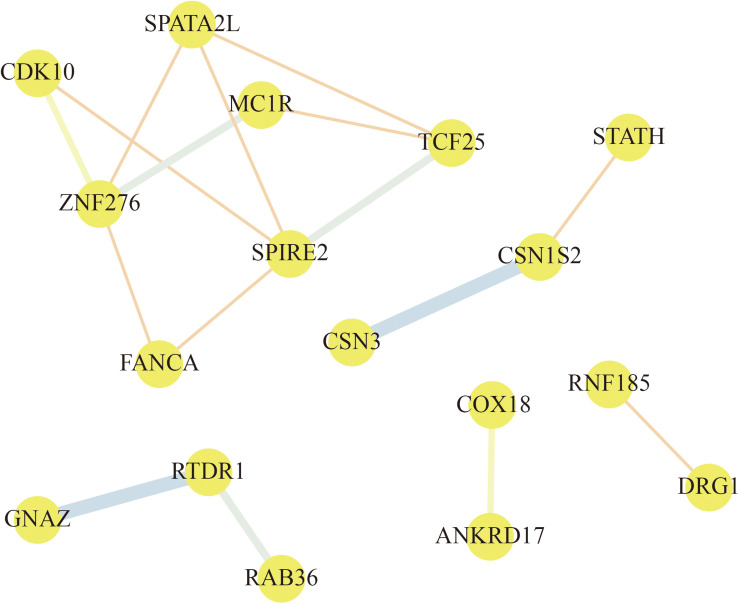
Protein–protein interaction (PPI) network of selected gene. The nodes represent proteins, and the line between the nodes indicate interactions between two connecting differentially expressed proteins (DEPs). The thickness of the line indicates the degree of interaction.

To explore the relationships among these genes, a PPI network of the selected genes was constructed by STRING and visualized by Cytoscape. According to the node pair combing score ≥0.4, a total of 17 selected genes were filtered into the PPI network that interacted with other selected genes (Figure 7). As shown in Figure 7, the PPI network of selected genes comprised 17 nodes and 16 edges. From this analysis of selected genes, we focused on the selected genes that interacted with three or more other genes. ZNF276, SPIRE2, TCF25, and SPATA2L are found to be the hub genes in the network. Based on these results, we assumed that ZNF276, SPIRE2, TCF25, and SPATA2L could be promising candidate genes that affect “cellular process,” “metabolic process,” “cell,” “macromolecular complex,” etc., indicating that these genes might play an important role in metabolism and cell process.

## Discussion

As one of the first domesticated animals, pig has played an important role in many aspects of human life. The ancestors of domesticated pig present in the world provide a unique opportunity for elucidating the genetics basis of domestication and further promoting the breeding of pig. In recent years, selection signatures have been identified in agricultural animals, leading to the elucidation of the mechanisms of many complex traits. To better understand the genetic basis underlying domestication and natural selection, we performed whole-genome resequencing on 20 unrelated ASP combined with 13 downloaded Asian wild boars. We selected windows with simultaneously high *F*_*ST*_ values (1% right tail) and significantly high θπ ratios (1% right tail). There are 157 selected regions with 48 genes harbored in these regions. Functional enrichment analyses revealed that the selected genes may play an important role in reproduction, immune system process, growth, salivary secretion, coat color, and other traits.

We found that a gene could elucidate the genetic basis of coat color phenotype of ASP population. In previous studies about pig coat color, *MC1R* gene was under positive selection (Li et al., 2010; Zhao et al., 2018). In this study, the *MC1R* gene was also found under selection based on cross approaches. To determine whether the six-end-white phenotype in the ASP is associated with the 2-bp insertion in the coding sequence of the *MC1R* gene, as previously shown in Bama miniature pigs (Jia et al., 2017), we searched for the presence of a 2-bp insertion in *MC1R* not detected in ASP population. This observation implies that the six-end-white phenotype in the ASP is not caused by a 2-bp insertion in the *MC1R* gene. We found five missense variants (exon1: c. G283A: p.V95M; exon1: c.T305C: p. L102P; exon1: c. A727G: p. T243A; exon1: c. T491C: p.V164A; exon1: c. G364A: p.V122I) within the *MC1R* gene. The amino acids changed by G283A and T503C were identified in ASP but absent from wild boars, which is consistent with a previous study (Li et al., 2010). Meanwhile, the “exon1: c.T305C: p. L102P” variant could result in a Leu to Pro substitution. Evidence from other species strongly suggest that the change from a Leu at codon to a Pro substitution was associated with a dominant black color trait (Klungland et al., 1995; Kijas et al., 1998). These results further imply that the ASP coat color is likely attributed by *MC1R* mutations Therefore, the identification in *MC1R* helps us to better explain the black coat of the ASP breed. Besides these well-known loci, further investigation about the effect of other missense variants on coat color phenotypic variation in ASP needs to be done. The putative role of these variants should be tested with functional experiments.

We also found a selected gene related to “salivary secretion.” Saliva played an important role as lubricant and an antimicrobial, preventing the dissolution of teeth, aiding in digestion, and facilitating taste (Carpenter, 2013). In a previous study, it has been proven that domestic pig produces more saliva than Tibetan wild boars and found that *KCNMA1* and *TRPC1* exhibited strong selective signals (Li et al., 2013). Even though the two genes were not harbored in the selection regions, we also found a gene Statherin (*STATH*) related to “salivary secretion” pathway, which has been under selection pressure in ASP. Statherin is a salivary protein encoded by the *STATH* gene, which helps to control the formation of hydroxyapatite crystals and plays an important role in maintaining the tooth enamel in the oral cavity (Schwartz et al., 1992; Goobes et al., 2006). The *STATH* gene is expressed in the human, dog, and pig salivary gland (Schlesinger and Hay, 1977; Nakanishi et al., 2016), and it was confirmed that the messenger RNA (mRNA) of the *Statherin* (*STATH*) gene was present in saliva but absent in blood, semen, vaginal secretions, and menstrual blood (Tsai et al., 2018). Positive selection of the *STATH* gene is consistent with the observation that domestic pig produces more saliva than wild boars (Li et al., 2013).

As is well known, the domestic pigs have a higher fertility than wild boars. We identified several genes involved in GO term related to reproduction. They played an important role in asymmetry, spermatogenesis, embryo cleavage and blastocyst formation, meiosis and germ cell development, and acrosome reaction. Of these genes, spire type actin nucleation factor 2 (*SPIRE2*) was found playing a key factor in asymmetric division of mouse oocytes, and the mRNA levels of *SPIRE2* in oocytes are significantly higher than in other tissues (Pfender et al., 2011). Meanwhile, the asymmetric oocyte division is essential for fertility (Leader et al., 2002). In previous studies, oxysterol-binding protein 2 (*OSBP2*) has been elucidated playing an important role in the postmeiotic differentiation of germ cells and would cause male infertility owing to oligo-astheno-teratozoospermia with lack of *OSBP2* (ORP4) (Charman et al., 2014; Udagawa et al., 2014). This may imply that *OSBP2* is significantly associated with spermatogenesis. *LIM kinases 2* (LIMK2), especially the testis-specific isoform tLIMK2, is specifically expressed in differentiated, meiotic stages of spermatogenic cells and plays an important role in proper progression of spermatogenesis by the regulation of cofilin activity and/or localization in germ cells (Takahashi et al., 2002). The weight of the testes in LIMK2−/− mice was significantly reduced to approximately 80% compared to that of control mice (Takahashi et al., 2002). In addition, the inhibition of LIMK1/2 activity in mouse causes the failure of embryo cleavage and blastocyst formation (Duan et al., 2018). Fanconi anemia (*FANCA*) genes, traditionally known for their essential roles in DNA repair and cytogenetic instability, have been demonstrated to be involved in meiosis and germ cell development. In a previous study about premature ovarian insufficiency (POI), two missense variants of *FANCA* were identified and could reduce its protein expression level compared with non-POI women. Meanwhile, heterozygous mutated female mice (Fanca±) showed reduced fertility and declined numbers of follicles with aging when compared with the wild-type female mice (Yang et al., 2019). Calcium-binding protein, sperm-specific 1 (*CABS1*) was first reported as one of the genes highly expressed in human testis (Jiahao et al., 2000) and specifically expressed in mice in the elongate spermatids and then localized into the principal piece of flagella of matured spermatozoa (Kawashima et al., 2009). Shawki et al. found that the porcine *CABS1* localizes to the acrosome in addition to the tail where mCABS1 only localizes in mature sperm, suggesting that porcine *CABS1* is involved in the acrosome reaction (Shawki et al., 2016).

In a previous study, it has been reported that the larger and more diverse olfactory gene repertoire may help pig to recognize odors that disseminated from a wide range of food types and flavoring agents that are present in artificial feeds, which result in higher feed intake and pork yield in duroc pig (Nguyen et al., 2012). It is well known that the feed intake and pork yield of duroc are higher than that of Chinese domesticated pigs. On the other hand, when comparing to wild boars, the Chinese domesticated pigs had a higher feed intake and pork yield, which may result from the olfactory genes in domesticated pigs. In this study, a gene (*OR5K4*) in ASP was selected and related to “olfactory transduction,” we thus assume that selection on specific olfactory receptors could enable domestic pigs to have a higher feed intake and pork yield. This may explain the signatures of selection at the olfactory receptor gene and the Chinese domesticated pigs having a higher feed intake and pork yield than wild boars.

Several genes related to “growth” were also identified. Neuropeptide Y (NPY) is widely expressed in the central nervous system and influences many physiological processes, including food intake (Zarjevski et al., 1993) and bone density (Teixeira et al., 2009). Meanwhile, Y1 and Y5 receptors (*NPY1R* and *NPY5R*) are expressed in hypothalamic areas that control feeding (Larsen et al., 1993; Parker and Herzog, 1999). It has been demonstrated that both *NPY1R* and *NPY5R* played a key role in the control of food intake in mice (Raposinho et al., 2004). Selenoprotein M (*SELENOM*), a positive regulator of leptin signaling and thioredoxin antioxidant activity in the hypothalamus, has been demonstrated to play a key role in Ca^2+^ homeostasis and energy metabolism (Gong et al., 2019). Mice with Selenom−/− knockout exerted a significant influence on energy homeostasis, including an increased body weight and reduced hypothalamic leptin sensitivity (Pitts et al., 2013).

Although some interesting findings were reported here, the limitations of the present study should not be neglected. On the one hand, the alleles were described from a limited sample size of ASP (*n* = 20) and wild boars (*n* = 13), which might not completely represent the populations and affect the *F*_*ST*_ and θπ ratio statistic. On the other hand, the function of these selected genes was annotated with GO and KEGG database, and although studies have been done, there is still a need to conduct investigations to understand the underlying genetic mechanism on traits in pig. The limitations might impact the observations of this study and should be overcome in further investigations.

This study detected the genomic signatures of selection that may have shaped the domestication of ASP in China. The genes found to be positively selected in ASP are involved in crucial biological processes such as coat color (*MC1R*), salivary secretion (*STATH*), reproduction (*SPIRE2*, *OSBP2*, *LIMK2*, *FANCA*, and *CABS1*), olfactory transduction (*OR5K4*), and growth (*NPY1R*, *NPY5R*, and *SELENOM*). In addition, mutations within these genes were also identified, which can be used to further refine selection in ASP in the future. Our research increased the knowledge of ASP phenotype-related genes and helped to improve our understanding of the underlying biological mechanisms.

## Data Availability Statement

The datasets presented in this study can be found in online repositories. The names of the repository/repositories and accession number(s) can be found in the article/Supplementary Material.

## Ethics Statement

The animal study reviewed and approved in this study was carried out in accordance with the recommendations of the Animal Care Committee of Anhui Academy of Agricultural Sciences (Hefei, China). The protocol was approved by the Animal Care Committee of Anhui Academy of Agricultural Sciences (No. AAAS2020-04).

## Author Contributions

WZ, CW, and ZY: conceptualization. WZ, MZ, MY, YW, XW, XZ, and YD: data curation. WZ: writing—original draft preparation and project administration. WZ, CW, ZY, and GZ: writing—review and editing. CW and ZY: funding acquisition. All authors have read and agreed to the published version of the manuscript.

## Conflict of Interest

The authors declare that the research was conducted in the absence of any commercial or financial relationships that could be construed as a potential conflict of interest.

## Footnotes

1 https://github.com/BGI-shenzhen/PopLDdecay

2 www.animalgenome.org

3 http://string-db.org/

4 http://www.cytoscape.org/

5 ftp://ftp.ensembl.org/pub/release-99/variation/gvf/sus_scrofa/
